# Arsenic Poisoning Successfully Treated by Dialysed Iron

**Published:** 1878-05

**Authors:** 


### Another Case of Arsenical Poisoning Successfully Treated by Dialyzed Iron.

James Hayes, M. D., C. M., Simcoe, Ontario, in the March number, 1878, of the *Canada Lancet*, says that during the evening of November 14th, 1877, he was summoned by Mrs. B.'s char-woman who had accidentally taken arsenic. Mrs. B. had purchased some arsenious acid with which to destroy mice. During the morning of November 14th, she had spread at least a half teaspoonful of the poison upon a slice of buttered bread, and placed it on a shelf in the pantry. During her absence from home for a short time, the char-woman went into the pantry and, not being aware there was any poison upon the bread, ate the whole. She stated she thought it was rather gritty. On Mrs. B.'s return a few minutes after, the woman complained of being sick, with cramps in her stomach, and wished some ginger tea to relieve them. Mrs. B. went to the pantry for the ginger, when she found the poisoned bread gone. On asking the woman, Mrs. B. was horrified to learn that she had eaten it.

I administered an emetic and promoted vomiting by large draughts of warm water. After the stomach had been thoroughly emptied, I gave a tablespoonful of dialyzed iron, diluted with water, which was rejected in a few minutes. I then repeated it in thirty drop doses every twenty minutes for two hours, and afterwards at longer intervals. About two hours after my arrival, alarming symptoms of collapse showed themselves; the pulse became extinct at the wrist; the skin cold and clammy, etc.; but by giving brandy freely, with the application of hot bottles and friction, she began to revive, and went on gradually improving until, in about ten days, she appeared to be restored to her accustomed good health. The only unpleasant symptoms she complained of during her convalescence were weakness, thirst and a burning sensation in the stomach.

I attribute this woman's recovery entirely to the dialyzed iron. It appears almost incredible that recovery should have taken place, considering the amount of arsenious acid swallowed. There must have been fully half a teaspoonful of acid, which was lying in the stomach from half an hour to one hour before I saw her.—*Virginia Medical Monthly.*

